# Relative Risk of All‐Cause Mortality Associated With Cannabis Use: A Systematic Review and Meta‐Analysis of Cohort Studies

**DOI:** 10.1002/hsr2.71212

**Published:** 2025-09-26

**Authors:** Zainab Alimoradi, Chung‐Ying Lin, Daniel T. Myran, Marco Solmi, Amir H. Pakpour

**Affiliations:** ^1^ Social Determinants of Health Research Center, Research Institute for Prevention of Non‐Communicable Diseases Qazvin University of Medical Sciences Qazvin Iran; ^2^ Institute of Allied Health Sciences, College of Medicine National Cheng Kung University Tainan Taiwan; ^3^ Biostatistics Consulting Center, National Cheng Kung University Hospital, College of Medicine National Cheng Kung University Tainan Taiwan; ^4^ School of Nursing, College of Nursing Kaohsiung Medical University Kaohsiung Taiwan; ^5^ Clinical Epidemiology Program Ottawa Hospital Research Institute Ottawa Ontario Canada; ^6^ Department of Family Medicine University of Ottawa Ottawa Ontario Canada; ^7^ Bruyère Health Research Institute Ottawa Ontario Canada; ^8^ Scientist North York General Hospital Toronto Ontario Canada; ^9^ SCIENCES Lab, Department of Psychiatry, Faculty of Medicine University of Ottawa Ontario Canada; ^10^ Department of Mental Health The Ottawa Hospital Ontario Canada; ^11^ Ottawa Hospital Research Institute (OHRI) Ottawa Ontario Canada; ^12^ Department of Child and Adolescent Psychiatry Charité Universitätsmedizin Berlin Germany; ^13^ School of Epidemiology and Public Health, Faculty of Medicine University of Ottawa Ottawa Canada; ^14^ Department of Nursing, School of Health and Welfare Jonkoping University Jonkoping Sweden

**Keywords:** cannabis use, cohort study, meta‐analysis, mortality, systematic review and meta‐analysis

## Abstract

**Background and Aims:**

Cannabis use has high prevalence and health burden. Although the effects of cannabis use have been studied in the literature, no systematic review and meta‐analysis has measured its association with all‐cause mortality. The aim of this systematic review and meta‐analysis was to systematically synthesize the evidence on association between cannabis use and all‐cause mortality.

**Methods:**

Following the preregistered protocol (PROSPERO: CRD42023396915), we searched in Scopus, PubMed, Web of Science and ProQuest databases until end of October 2023. We included cohort studies comparing individuals using versus not using cannabis and measuring the association with all‐cause mortality. A random‐effect meta‐analysis was conducted calculating the risk ratio (RR) and 95% confidence interval (CI). Heterogeneity and publication bias were measured. Sensitivity and meta‐regression analyses were conducted. The Newcastle Ottawa Scale (NOS) was used to assess study quality.

**Results:**

Fourteen cohort studies were included (prospective 50%), reporting on 17,545,076 participants (3,000,667 people who use cannabis [PWUC]). The overall RR estimation of all‐cause mortality among PWUC versus nonusers was 1.53 (95% CI: 1.09; 2.14, *I* ^2^: 98%; *τ* ^2^: 0.38). Significantly different RR was observed in prospective versus retrospective designs (2.07 vs. 1.11); cohorts of the general population versus patients (2.53 vs. 1.03). Study sample size was a significant moderator of the association between cannabis use and all‐cause mortality, with larger sample size being associated with smaller effect size and less heterogeneity. Based on GRADE assessment, observational evidence, with unadjusted estimates, high heterogeneity with inconsistent results, the overall certainty of evidence seems to be low.

**Conclusion:**

Cannabis use was associated with an increased risk of all‐cause mortality in the general population but not in patients with severe underlying medical co‐morbidities. It should be noted that the evidence may currently be biased and new methodologically strong studies need to be conducted.

## 1 Introduction

Cannabis is the third most used psychoactive substance in the world after alcohol and tobacco [1, 2, 3]. In 2019, an estimated 23.8 million (38.6% of age‐standardized prevalence) people worldwide have used cannabis according to the Global Burden of Disease 2019 [4]. Emerging evidence suggests that cannabis legalization may be one factor influencing rising prevalence rates, though observed trends began before legalization in some regions [5, 6]. Reported increases could reflect both actual changes in use and improved disclosure due to reduced stigma [7]. However, concurrent factors including shifting social norms, commercialization, and pre‐existing use patterns complicate causal attribution.

Based on a recent systematic review and meta‐analysis which pooled evidence from 2000 to 2024, there was a geographical difference in pooled prevalence and its' trend in cannabis use. They reported varied prevalence of cannabis use between 0.42% and 43.90% across 33 European countries, 1.40% to 38.12% across 15 North and South American countries, 0.30% to 19.10% across 16 Asian countries, and 1.30% to 48.70% across 18 Oceania and African countries. The pooled prevalence of cannabis use was 12.0% [95% confidence interval (CI): 10.0, 14.3] in countries where cannabis is legalized, compared to 5.4% (95% CI: 4.3, 6.9) in non‐legalized countries [8]. Cannabis use exists on a spectrum, ranging from occasional recreational use to chronic or dependent use [1, 9]. While present systematic review with meta‐analysis focuses on the association between cannabis use and all‐cause mortality, it is critical to recognize that not all cannabis use leads to adverse outcomes. The severity and frequency of use, as well as individual susceptibility, play significant roles in determining health consequences [10].

The association between cannabis use and increased mortality may not have a direct causal link between cannabis use and death; instead, the association may be mediated through several pathways [11]. First, cannabis use, particularly heavy or prolonged use, has been linked to psychiatric disorders such as schizophrenia, depression, and psychosis, which in turn elevate suicide risk [12]. Second, acute cannabis intoxication impairs cognitive and motor functions, increasing the likelihood of fatal traffic accidents [13]. Third, chronic use may contribute to cardiovascular and respiratory complications, though evidence remains inconsistent [14]. These mechanisms suggest that cannabis‐related mortality is likely multifactorial, involving both direct and indirect effects.

Cannabis use causes health burdens globally [6]. While cannabis use exists on a spectrum from occasional recreational use to problematic dependence, evidence suggests that regular or heavy non‐prescribed use, particularly when beginning in adolescence, is associated with increased health risks globally [10, 15]. The greatest health burdens appear concentrated among individuals with cannabis use disorders or those using high‐potency products, while occasional adult use may present minimal risk for most users [16]. These differential impacts underscore the importance of distinguishing between use patterns when assessing population‐level health consequences. Empirical evidence indicates that chronic, heavy cannabis use particularly of high‐THC products is associated with functional impairments, including: cognitive disabilities (e.g., memory and attention deficits, especially with adolescent‐onset use [17]; psychiatric morbidity (e.g., exacerbation of psychotic disorders [15]; and physical health limitations (e.g., cannabis hyperemesis syndrome, respiratory symptoms in chronic smokers [18]. These disability outcomes are primarily observed at the severe end of the use spectrum (≥ 4 days/week), with minimal functional impact observed among occasional adult users [16]. Recent epidemiologic evidence indicates that the disability‐adjusted life years (DALYs; an index indicating years lost due to disability in a cumulative calculation) for people with cannabis use disorders were 0.69 million [6]. Moreover, data from the Global Burden of Disease Study revealed that the populations at greater risk of cannabis use are men, those living in high‐income countries, and those with high incomes [6, 9, 15]. The reasons for cannabis use are multifaceted and influenced by a combination of biological, psychological, and social factors. Peer pressure, cultural norms, stress relief, and self‐medication for mental or physical health conditions are among the key drivers of use [19, 20]. Understanding these contextual influences is essential for developing targeted interventions and policies.

Although there are substantial differences in the prevalence of cannabis use across different demographic and geographical features, an increasing trend of burden caused by cannabis use has been observed. For example, the global DALYs increased 38.6% in a period of 30 years from 1990 to 2019 [6]. The health and economic burdens associated with problematic cannabis use include: mental health impacts (e.g. increased risk of psychosis disorders [especially in heavy adolescent users], cannabis use disorder, and exacerbation of mood disorders); physical health costs due to cannabis‐related emergency department visits (e.g., for hyperemesis syndrome) and respiratory complications in chronic smokers; economic consequences due to lost productivity and healthcare utilization costs; and social burdens due to impaired driving accidents and associated legal system costs [6, 9, 21].

In this regard, addressing the issue of cannabis use prevention is urgent and important. Cannabis remains prohibited in most countries under international drug control treaties, though policy rationales are multifaceted. While health concerns (e.g., psychosis risk, driving impairment) contribute to these policies [15], historical factors (e.g., racialized prohibition campaigns), economic interests (e.g., alcohol/tobacco industry opposition), and moral stigmatization have equally shaped criminalization [7, 22, 23]. This complex legacy persists despite evidence that decriminalization does not necessarily increase population‐level harm [24]. Beyond its health implications, cannabis use is often stigmatized, which can exacerbate social and psychological burdens for users [7]. Stigmatization may discourage individuals from seeking help for problematic use, worsen mental health outcomes, and perpetuate marginalization. Addressing stigma is crucial for fostering equitable public health approaches [25].

While this review highlights the risks associated with cannabis use, it is important to acknowledge its potential benefits in certain contexts. The evidence on the therapeutic potential of cannabidiol or tetrahydrocannabinol (THC) in a small number of selected populations with specific conditions (e.g. skin health and disorders [26], seizure disorders, pain, muscle spasticity, stimulating appetite, and treating nausea/vomiting [16]) had been reported. However, these benefits must be weighed against the broader public health implications, particularly for recreational or nonmedical use.

Current evidence indicates that cannabinoids largely have detrimental effects on human health, as recently summarized in large scale umbrella review [15]. Problematic cannabis uses particularly heavy, long‐term, or adolescent‐onset consumption has been associated with increased risks of schizophrenia spectrum disorders, motor vehicle accidents, and suicidal behaviors [15, 22]. Also some emerging evidence that may increase risk of cerebrovascular disease and possibly cancer [18]. For cerebrovascular disease, meta‐analyses of cohort studies report modest but significant hazard ratios (1.15–1.42) for ischemic stroke among daily users, particularly in younger populations (< 45 years), though these findings may be confounded by concurrent tobacco use and other vascular risk factors [27, 28]. Regarding cancer risk, systematic reviews have identified statistically significant but clinically modest associations for testicular germ cell tumors (summary OR = 1.30, 95% CI: 1.08–1.56) and limited evidence for lung cancer primarily among cannabis smokers who also use tobacco [18]. These associations likely reflect a combination of pharmacological effects, pre‐existing vulnerabilities, and socioeconomic confounders rather than direct causation [29]. When present concurrently, these factors may contribute to elevated all‐cause mortality observed in some populations [9].

Collectively, these associations suggest that heavy, chronic cannabis use may contribute to increased all‐cause mortality risk, though the only systematic review on this topic was published fifteen years ago [30]. This evidence gap is particularly critical given: (1) rising global cannabis consumption following policy liberalization [6], (2) increased THC potency in contemporary products [31], and (3) persistent methodological limitations in existing studies (e.g., inadequate adjustment for polysubstance use). However, this gap primarily reflects underinvestment in longitudinal research rather than definitive population‐level health impacts.

Despite the large number of meta‐analyses published on the associations between cannabis and health outcomes, only one prior systematic review and meta‐analysis published fifteen years ago examined the association between cannabis use and death [30]. Since then, numerous additional cohort studies have measured whether persons using cannabis suffer from a life expectancy gap compared to those who do not [30, 32, 33]. The present systematic review and meta‐analysis aimed to systematically evaluate cohort studies investigating the relationship between cannabis use and all‐cause mortality. For clarity, ‘all‐cause mortality’ refers to death from any cause, encompassing both natural and unnatural fatalities [34]. This systematic review synthesizes evidence from cohort studies to assess the association between cannabis use and mortality, while the meta‐analysis quantifies this relationship through pooled risk estimates. Systematic reviews rigorously evaluate existing literature, whereas meta‐analyses employ statistical methods to combine results across studies, enhancing the precision and generalizability of findings.

### 1.1 Systematic Review Question

In the general and clinical populations, does cannabis use (compared to no use) increase the risk of all‐cause mortality?

## 2 Methods

### 2.1 Protocol Registration

The study protocol of the present systematic review and meta‐analysis was registered on the PROSPERO website (CRD42023396915) [35].

### 2.2 Information Sources

Four academic databases, namely Scopus, PubMed, Web of Science and ProQuest, were systematically searched from inception to October 2023. To increase the possibility of including relevant papers, the reference lists of the included studies were manually searched to retrieve studies that were not captured by the search strategy. Additional manual searches were conducted in Google Scholar using the same key terms adapted for database searches (e.g., [“cannabis” AND “mortality”]). The top 100 results sorted by relevance were screened for eligibility.

### 2.3 The Eligibility Criteria

The systematic review question and eligibility criteria followed the PECO framework [36] as below:

- Participants: People with cannabis use (i.e., PWUC) without demographic restrictions.
- Exposure: Any cannabis use.
- Comparator: No cannabis use.
- Outcome: All‐cause mortality.

Study design (prospective or retrospective cohort studies) was applied as an additional inclusion criterion during screening. Prospective cohort studies identify cannabis exposure at baseline and follow participants forward in time to assess mortality, minimizing recall bias. Retrospective cohort studies use pre‐existing records to classify exposure and outcomes after they have occurred, offering logistical advantages but potentially introducing misclassification. Our analysis included both designs to balance methodological strengths of prospective studies' temporal clarity and retrospective studies' large sample sizes. Language was restricted to English. Studies with the use of multiple substances were excluded. Studies published between 1 January 1990 and 31 October 2023 to capture patterns of cannabis use and associated mortality risks.

### 2.4 Search Strategy

The search strategy was developed by the study team (ZA, AHP) in consultation with a medical information specialist from Qazvin University of Medical Sciences' biomedical library to ensure comprehensive coverage. The specialist reviewed all search syntaxes for proper MeSH/Emtree term utilization and Boolean logic. The search was performed using keywords from relevant MeSH terms. In the study, key terms related to two components of exposure (Cannabi* OR Plant* OR Hemp* OR Marihuana OR Marijuana OR “Cannabis indica” OR “*Cannabis sativa*” OR Hashish* OR Bhang* OR Ganja*) and outcome (mortalit* OR Death) was selected to develop search syntax. Database search strategies use a combination of the following keyword sets within the titles, abstracts, and keywords. For manual search in Google Scholar, the adapted terms of (“cannabis use” OR “marijuana”) AND (“mortality risk” OR “death rate”) were used. The search syntax was adapted for other databases. Sample search syntax for PubMed is provided in Supplement file 1.

### 2.5 Screening

Following the Preferred Reporting Items for Systematic Reviews and Meta‐Analyses (PRISMA) guidelines 2020 [37], a two‐step process of screening and selection was used. First, the titles and abstracts of all the retrieved studies were scrutinized for suitability based on the eligibility criteria. Then, full texts of potentially relevant studies were thoroughly read to assess the possibility of final inclusion. The screening processes were independently conducted by two reviewers. All disagreements were resolved by consensus.

### 2.6 Study Selection

Studies that met the eligibility criteria and reported relevant data to calculate the risk of all‐cause mortality in association with cannabis use were included for data collection. Studies were considered to have reported relevant data if they contained: quantitative measures of association between cannabis use and all‐cause mortality (e.g., risk ratios, hazard ratios, or raw data to calculate these); sample sizes for both exposed (people who use cannabis [PWUC]) and unexposed groups; duration of follow‐up and demographic characteristics (age, sex distribution). Studies were excluded if they did not explicitly document mortality ascertainment methods (e.g., source of death data, verification procedures. For causal interpretation, we prioritized studies with prospective measurement of cannabis exposure (preceding mortality). In studies which report subgroups of different substances use, only cannabis subgroup was considered for data extraction, and no substances use group was considered for comparator. The study selection processes were independently conducted by two reviewers. All disagreements were resolved by consensus.

### 2.7 Data Collection and Data Items

For data collection, a predefined Excel spreadsheet was used. The following data were extracted for the present study: author(s), cohort design, publication date, study beginning and end dates, country and income level with development status based on the latest World Bank report, population, follow‐up duration, sample size, sex proportion, age range with mean, and data from 2*2 tables to calculate the risk ratio (RR).

***N.B.*** It is important to note that different effect sizes include Odds Ratio (OR), Risk Ratio (RR) and Hazard Ratio (HR). Due to this variation in reported effect size, RR was selected effect size for current systematic review and meta‐analysis which raw data was reported in most eligible studies. The data collection processes were independently conducted by two reviewers. All disagreements were resolved by consensus.

### 2.8 Assessment of Study Risk of Bias

The risk of bias within the included studies was assessed using the Newcastle–Ottawa Scale (NOS) for cohort studies. Three domains of selection (four items), comparability (one item), and outcome assessment (three items) were assessed. A study can be awarded a maximum of one star for each numbered item within the selection and outcome domains, and a maximum of two stars can be given for comparability [38]. “Good quality” is defined as 3 or 4 stars in the selection domain AND 1 or 2 stars in the comparability domain AND 2 or 3 stars in the outcome/exposure domain; Fair quality is defined as 2 stars in the selection domain AND 1 or 2 stars in the comparability domain AND 2 or 3 stars in the outcome/exposure domain; and Poor quality is defined as 0 or 1 star in the selection domain OR 0 stars in the comparability domain OR 0 or 1 stars in the outcome/exposure domain [39]. Overall, studies were categorized as low risk of bias when scoring above five points [40]. Two reviewers independently assessed the risk of bias. All disagreements were resolved by consensus.

### 2.9 Effect Measures

The risk ratio (RR) and its 95% confidence interval (CI) were selected as measures of the effect in the present systematic review and meta‐analysis. To calculate RRs, raw data from 2*2 tables were extracted from the included studies.

### 2.10 Synthesis Methods

The evidence extracted from the included studies was synthesized quantitatively using meta‐analysis. Quantitative synthesis was performed using Comprehensive Meta‐Analysis (CMA) software, version 3.0 (Biostat, Englewood, NJ, USA). This software employs random‐effects models to account for between‐study heterogeneity, and provides tools for subgroup analysis, meta‐regression, and assessment of publication bias through funnel plots and Egger's test. CMA was selected for its capability to handle diverse effect size metrics (RR, OR, HR) and its rigorous algorithms for heterogeneity estimation (I² and τ²), which are essential for our study design. Analyses followed the software's guidelines for complex meta‐analyses [41]. Random effect models estimating within‐ and between‐study variances were used to consider heterogeneity among different populations [42]. To assess possible sources of heterogeneity and influential moderators, subgroup analysis (for variables of cohort design, geography, target population) or meta‐regression (for variables of mean age, sex distribution, length of follow‐up, sample size) was conducted.

### 2.11 Assessment of Reporting Bias

Egger's test and funnel plots were used to assess the probability of publication bias [43]. The probability of a small study effect was assessed using the jackknife (or one‐out) method [44].

### 2.12 Certainty Assessment

The certainty of evidence was assessed using the GRADE approach, a rating system for assessing the quality of evidence in systematic reviews and other evidence syntheses [45]. The GRADE evidence profile contains a summary of findings for outcomes as well as detailed information about the quality of evidence based on study design and participants, risk of bias, inconsistency, indirectness, imprecision, and publication bias [46].

## 3 Results

### 3.1 Study Selection

**Figure 1:**
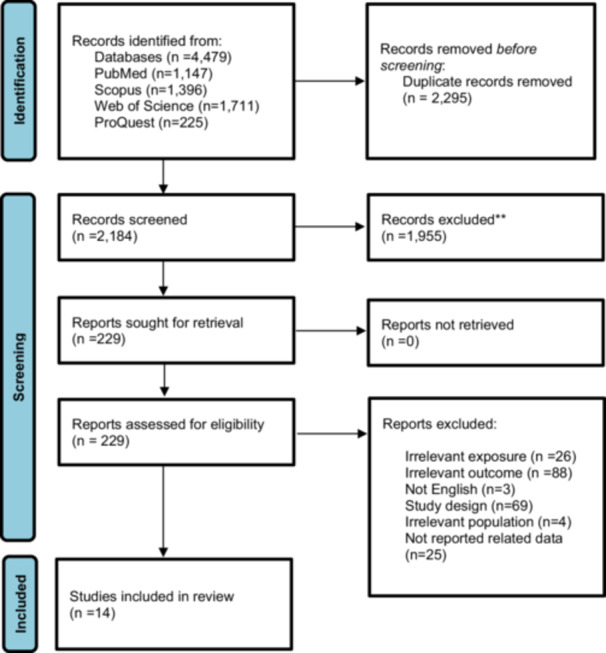
PRISMA flowchart showing the search, selection, and analysis process.

Overall, 4,479 papers were retrieved through the search strategy (PubMed (*n* = 1,147), Scopus (*n* = 1,396), Web of Science (*n* = 1,711), ProQuest (*n* = 225), in which 2295 papers were duplicates and removed. The titles and abstracts of the remaining papers (*n* = 2184) were screened. Finally, 14 studies were included in the present systematic review and meta‐analysis. Manual search in reference list of included studies and Google Scholar did not lead to retrieving any new paper. The PRISMA flowchart (Figure 1) shows the search, selection, and analysis process.

### 3.2 Study Characteristics

The 14 included studies were all from developed and high‐income countries (7 from the USA, 2 from Canada, 3 from Sweden, 1 from Israel, and 1 from France), involving 17,545,076 participants (3,000,667 PWUC). The participants ranged in age from 10 to 80 years, with a mean age of 48.5 years (reported in 11 out of 14 studies), and the participants were predominantly (63.5%) males. The sample size varied between 1028 and 6,947,191 participants. All studies included cohorts with the same proportion (7 out of 14) of prospective and retrospective designs. The participants in the cohorts were from the general population in six studies and were patients with different medical problems (with details in Table 1) in the remaining studies. The summarized characteristics of the included studies are provided in Table 1.

**Table 1:** Summarized characteristics of included studies.

Author, year	Country	Study population	Study design	Start‐ end date of sampling	Follow‐up duration (year)	Sample size/female %	Age range	Age mean
Sidney et al., [47]	USA	General population	Prospective Cohort	1979–1985	5	65171/57	15–49	33
Frost et al., [48]	Israel	Patients (Survivors of Acute Myocardial Infarction)	Prospective Cohort	1989–1996	13	2097/23	NR	52
Mukamal et al., [49]	USA	Patients (Following Acute Myocardial Infarction)	Prospective Cohort	1989–1994	4	1913/31	NR	61
Reynolds et al., [50]	USA	Patients (Critically III Patients with Pneumonia)	Retrospective cohort	2010–2017	NR	167095/NR	NR	NR
McGuinness et al., [51]	Canada	Patients (After vascular surgery)	Retrospective cohort	2006–2015	NR	510007/39	18–75	62
Fontanella et al., [52]	USA	Patients (Youths with Mood Disorders)	Retrospective cohort	2010–2017	1	204780/65	10–24	17
Dandurand et al., [53]	USA	Patients (After aneurysmal subarachnoid hemorrhage)	Retrospective cohort	2016–NR	1	42394/57	NR	62
Goel et al., [54]	USA	Patients (Major Elective Surgeries)	Retrospective cohort	2006–2015	NR	27206/72	18–65	37
Santos et al., [55]	France	Patients (HIV/HCV Co‑infected Patients)	Prospective Cohort	2005–2014	NR	1028/30	NR	49
Manrique‐Garcia et al., [56]	Sweden	General population	Prospective Cohort	1955–2011	41	50373/0	NR	60
Vozoris et al., [32]	Canada	General population	Retrospective cohort	2009–2015	1	15202/42	12–65	39
Bohnert et al., [57]	USA	General population	Retrospective cohort	2006–2011	6	4863086/8	18–80	NR
Davstad et al., [58]	Sweden	General population	Prospective Cohort	1969–2004	35	48024/0	NR	NR
Crump et al., [59]	Sweden	General population	Prospective Cohort	2003–2016	13	6947191/51	NR	62

### 3.3 Risk of Bias in Studies

The details of risk of bias assessment based on the NOS checklist for cohort studies in each domain of selection, comparability, and outcome are presented in Table 2. Ascertainments of exposure in 6 out of 14 studies were done using self‐report measures, or no description was provided. The outcome of interest was not present at the beginning of the study in 7 out of 14 studies. The adequacy of follow‐up or loss to follow‐up was not clear in 7 out of 14 studies. Overall, considering the point that studies with total ROB score of five as high methodological quality, all included studies were ranked as high quality.

**Table 2:** Summarized result of risk of bias assessment within included studies.

Author, year	Selection					Comparability		Outcome			
	S 1	S 2	S 3	S 4	ROB status	C 1	ROB status	O 1	O 2	O 3	ROB status
Sidney S et al., 1997 [47]	*	*	—	*	Good	*	Good	*	*	—	Fair
Frost L et al., 2013 [48]	*	*	*	*	Good	*	Good	*	*	—	Fair
Mukamal KJ et al., 2008 [49]	*	*	*	*	Good	*	Good	*	*	—	Fair
Reynolds PM et al., 2022 [50]	*	*	*	—	Good	*	Good	*	*	*	Good
McGuinness B et al., 2020 [51]	*	*	*	—	Good	*	Good	*	*	*	Good
Fontanella CA et al., 2021 [52]	*	*	*	—	Good	*	Good	*	*	*	Good
Dandurand C et al., 2019 [53]	*	*	—	—	Fair	*	Good	*	*	*	Good
Goel A et al., 2019 [54]	*	*	*	—	Good	*	Good	*	*	*	Good
Santos M E et al., 2020 [55]	*	*	—	*	Good	*	Good	*	*	*	Good
Manrique‐Garcia E et al., 2017 [56]	*	*	—	*	Good	*	Good	*	*	*	Good
Vozoris NT et al., 2022 [32]	*	*	—	—	Fair	*	Good	*	*	—	Fair
Bohnert KM et al., 2017 [57]	*	*	*	—	Good	*	Good	*	*	—	Fair
Davstad I et al., 2011 [58]	*	*	—	*	Good	*	Good	*	*	—	Fair
Crump C et al., 2021 [59]	*	*	*	*	Good	*	Good	*	*	—	Fair

*Note:* S 1. Representativeness of the exposed cohort. S 2. Selection of the nonexposed cohort. S 3. Ascertainment of exposure. S 4. Demonstration that outcome of interest was not present at start of study. C 1. Comparability of cohorts based on the design or analysis. O 1. Assessment of outcome. O 2. Was follow‐up long enough for outcomes to occur. O 3. Adequacy of follow up of cohorts N.B. Please see methodological section (risk of bias assessment) regarding the star rating explanation.

### 3.4 Results of Syntheses

#### 3.4.1 Overall Estimation

**Figure 2:**
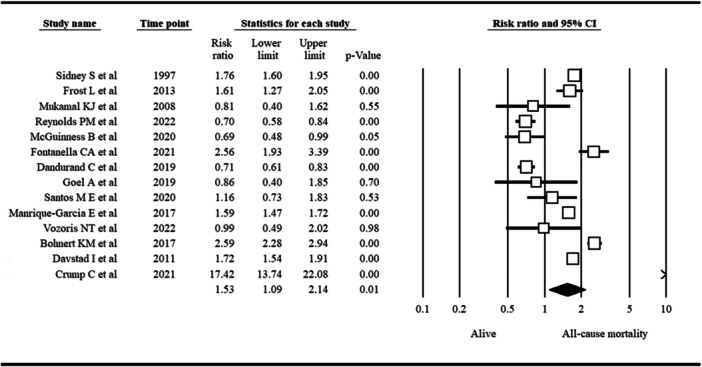
Forest plot showing the association between cannabis use and all‐cause mortality.

Overall RR estimation based on a meta‐analysis of 14 included studies revealed a significantly greater risk of all‐cause mortality in PWUC than in non‐ PWUC. The pooled RR was 1.53, with a 95% CI of 1.09 to 2.14 (*p* = 0.01; *I* ^2^: 98%; *τ* ^2^: 0.38). A forest plot showing the association between cannabis use and all‐cause mortality is presented in Figure 2.

#### 3.4.2 Publication Biases

**Figure 3:**
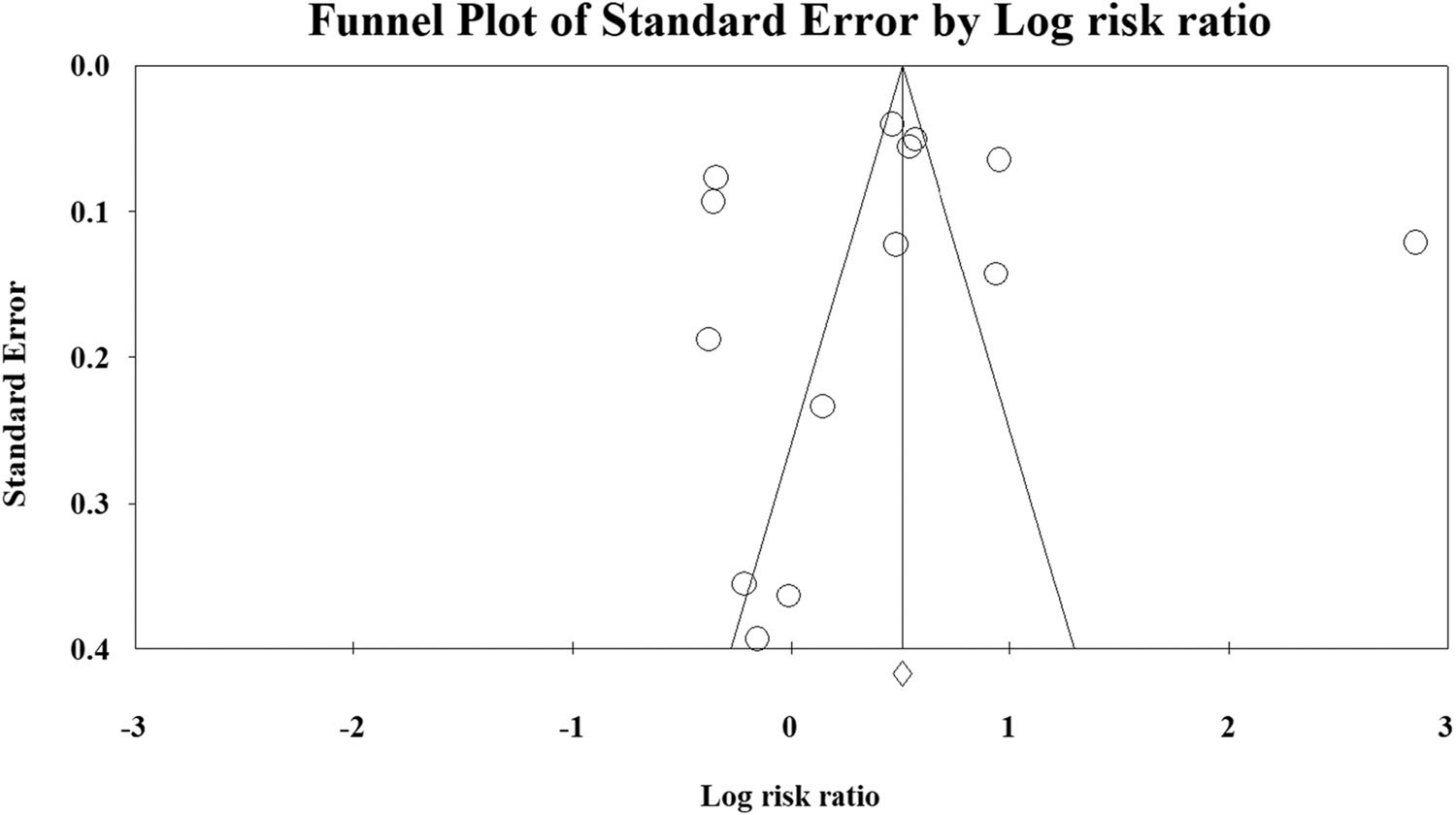
Funnel plot assessing the probability of publication bias in association with cannabis use and all‐cause mortality.

**Figure 4:**
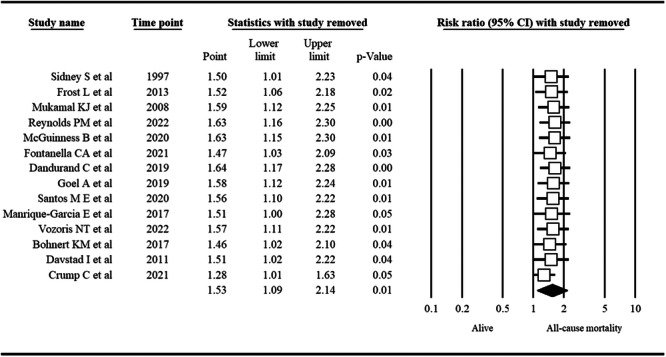
Sensitivity analysis assessing the small study effect on the association between cannabis use and all‐cause mortality.

The probability of publication bias was ruled out based on an insignificant Egger's test (*p* = 0.89) and a symmetric funnel plot (Figure 3). Sensitivity analysis revealed that the pooled effect size (i.e., RR) was not affected by the effect of a single study (Figure 4).

#### 3.4.3 Subgroup/Moderator Analysis

Subgroup analysis (Table 3) revealed significantly different results for prospective versus retrospective designs (RR [*p* for test of null]: 2.07 [0.02] vs. 1.11 [0.72]), cohorts of the general population versus patients (RR [*p* for test of null]: 2.53 [< 0.001] vs. 1.03 [0.88]). Methodologically, studies differed in the two domains of selection and outcome assessment. The influence of these methodological aspects on the pooled effect size was assessed using subgroup analysis. Studies with a good quality re selection bias showed higher RR (RR [95% CI]: 1.69 [1.20; 2.39]) than those with fair quality only (RR [95% CI]: 0.72 [0.62; 0.84]). Meta‐regression (Table 4) revealed that study sample size was a significant moderator (*p* = 0.01) of the association between cannabis use and all‐cause mortality, explaining 97% of the variance (RR decreased by increasing the sample size) and decreasing heterogeneity (67.44% vs. 98% in all study combinations).

**Table 3:** Results of subgroup analysis.

			No. of studies	RR (95% CI)	*p* value for test of null	*p* value for Interaction	*I* ^2^ (%)	Tau^2^
Cohort design		Prospective	7	2.07 (1.32; 3.25)	0.002	0.09	98.38	0.34
		Retrospective	7	1.11 (0.63; 1.98)	0.72		97.65	0.56
Population consisting cohort		General population	6	2.53 (1.60; 4.01)	< 0.001	0.003	98.70	0.31
		Patients	8	1.03 (0.71; 1.50)	0.88		92.66	0.25
Methodological domains	Selection bias	Good status	12	1.69 (1.20; 2.39)	0.003	< 0.001	97.98	0.34
		Fair status	2	0.72 (0.62; 0.84)	< 0.001		—	—
	Outcome assessment bias	Good status	7	1.06 (0.70; 1.59)	0.79	0.03	96.33	0.27
		Fair status	7	2.21 (1.33; 3.66)	0.002		98.32	0.43
All study combination			14	1.53 (1.09; 2.14)	0.01	—	98	0.38

**Table 4:** Results of univariable meta‐regression.

Variables	No of studies	Coefficient (log risk ratio)	Standard error	*p* value	*I* ^2^ (%)	Adjusted R^2^ (%)
Follow‐up duration (year)	11	0.006	0.03	0.85	98.56	−1.34
Study sample size	14	< 0.001	< 0.001	0.01	67.44	0.97
Percent of female participants	13	0.02	0.02	0.40	98.48	−1.17
Age mean of participants	11	0.001	0.02	0.68	98.65	−0.95

### 3.5 Certainty of Evidence

The GRADE approach was used to assess the certainty of evidence (Table 5). Overall, 14 cohort studies (7 prospective, 7 retrospective) comprising 17,545,076 participants (3,00,667 PWUC) were included in the present systematic review and meta‐analysis. All studies were high‐quality observational studies with a low risk of methodological bias. However, high heterogeneity led to inconsistency of results. Also selected effect size in current study is estimated based on unadjusted data, which led to serious indirectness based on participants, exposure or comparator's characteristics within the included studies. No imprecision was observed in the overall estimation, but imprecision in some subgroups was observed. No publication bias was found. The overall certainty of evidence seems to be low.

**Table 5:** Summary of findings and GRADE profile assessing certainty of evidence in association of cannabis use and all‐cause mortality.

Summary of finding		
PECO	Participants: Individuals using cannabis Exposure: Ever cannabis usage Comparator: Individuals not using cannabis Outcome: All‐cause mortality	
Pooled estimates	Risk ratio (95% CI): 1.53 (1.09; 2.14)	
Moderators	▪ Prospective versus retrospective designs ▪ Cohort of general population versus patients ▪ Studies' sample size	

Quality assessment		
Item	Explanation	Judgment [score assigned]
Study design and participants	14 cohort studies (7 prospective, 7 retrospective) comprising 17,545,076 participants (3,000,667 cannabis user)	High quality observational studies [−1 score]
Risk of bias	Low risk of bias based on NOS checklist for cohort studies (All studies scores above 5 in NOS checklist)	Low risk of bias [−0 score]
Inconsistency	High heterogeneity (*I* ^2^ = 98%) which studies' sample size found to be main source of (decreased *I* ^2^ to 67%)	High heterogeneity with identified source [−1 score]
Indirectness	Serious indirectness of results based on participants, exposure and comparator (all estimates were unadjusted)	Serious indirectness [−2 score]
Imprecision	No imprecision in overall estimation and subgroups with significant precise pooled estimation was identified (prospective designs/cohort of general population/and studies conducted in Europe). However, sill imprecision in some subgroups was observed.	No imprecision in overall estimation but imprecision in some subgroups [−1 score]
Publication bias	No publication bias was found	No publication bias [−0 score]
Overall quality of evidence	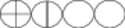	Low level of evidence

## 4 Discussion

Using the synthesized findings, the present systematic review and meta‐analysis estimated the association between all‐cause mortality and cannabis use in 17,545,076 participants (among whom 3,000,667 were PWUC) from 14 studies using a cohort design. The overall RR estimation was 1.53 (95% CI: 1.09, 2.14), indicating that PWUC, compared with cannabis nonusers, had a 53% greater chance of mortality. The findings could be considered robust given that the quality of all 14 studies was evaluated as fair to good across selection, comparability, and outcome domains using the NOS checklist (Table 2). Additionally, the GRADE approach indicates that the certainty of evidence is low. In addition to the overall RR estimation, subgroup analysis revealed several differences: (i) the prospective cohort design showed a significant RR for PWUC, but the retrospective cohort design did not; (ii) the general population had a significant RR for PWUC, but the patient population did not.

PWUC showed a 53% higher relative risk of mortality compared to nonusers (pooled RR = 1.53, 95% CI: 1.09–2.14). However, this association may reflect both direct toxic effects and residual confounding by unmeasured variables (e.g., polysubstance use, socioeconomic factors). The increased association between all‐cause mortality and cannabis use could be explained by the disability and impairments caused by cannabis use. Prior umbrella review findings showed that cannabinoids are associated with an increased risk of psychosis among the general population, as evidenced by both observational studies and randomized controlled trials [15]. Moreover, psychiatric symptoms (such as depression and mania) and cognitive impairments are associated with cannabis use [17], and motor vehicle accidents are associated with cannabis use [60]. Because these disabilities, impairments, and accidents can lead to mortality, PWUC are likely to have increased risks of mortality. While our analysis identifies associations between cannabis use and increased mortality risk, the causal pathways remain complex and multifactorial. Psychiatric symptoms (e.g., depression, psychosis) and cognitive impairments linked to cannabis use may contribute to mortality risk, but these relationships are likely bidirectional rather than unidirectional. For instance, individuals with pre‐existing depression may use cannabis as self‐medication, and their elevated suicide risk may stem primarily from the underlying mood disorder rather than cannabis use itself [61, 62]. Similarly, observed associations between cannabis use and accidental mortality may reflect confounding by polysubstance use or high‐risk behaviors rather than direct cannabis effects. These complexities underscore the importance of interpreting population‐level risk estimates cautiously, as they may capture true pharmacological effects of cannabis, consequences of comorbid conditions, or residual confounding by unmeasured variables. Nevertheless, the mechanisms underlying the relationship between mortality and cannabis use could be diverse, and future studies are needed to identify potential mechanisms involved.

When stratifying the included studies based on their study design, one could easily find that synthesized findings from retrospective cohort studies are nonsignificant. In contrast, the significant association between all‐cause mortality and cannabis use was strongly supported by synthesized prospective cohort studies. Therefore, it is important to identify why there was a large difference between prospective studies and retrospective studies. One potential reason is the common limitation of retrospective cohort studies regarding measurement bias. Specifically, both exposure (i.e., cannabis use in the present systematic review and meta‐analysis) and outcome measures (i.e., mortality in the present systematic review and meta‐analysis) are collected before the researchers conceptualize the research questions for a study. Therefore, the researchers could not use the measures they considered the most appropriate but could use only the existing collected data. In contrast, researchers who design prospective cohort studies could well‐prepare measures with reliable psychometric properties to assess both exposures and outcomes. Moreover, prospective cohort studies can train raters well before assessing exposure and outcomes, while some retrospective cohort studies (especially those in which data are collected simply for clinical purposes) may not have such training before data collection. Therefore, measurements in retrospective cohort studies might be less accurate than those in prospective cohort studies [63].

Subgroup analysis based on population type revealed divergent risk patterns: while general population studies showed significantly elevated mortality risk (RR = 2.53, 95% CI: 1.60–4.01), pooled results from patient populations were nonsignificant (RR = 1.03, 95% CI: 0.71–1.50). This contrast persisted across clinical subgroups, with psychiatric patient cohorts demonstrating marginally higher (albeit nonsignificant) risk (RR = 1.21, 95% CI: 0.95–1.54). The null association in patient groups may reflect competing mortality risks from underlying conditions, potential therapeutic monitoring effects in clinical settings, or survival bias where only healthier patients maintain long‐term cannabis use. Therefore, it is important to identify why there was a large difference between these two populations. A potential reason is the poor health conditions of the patients. Specifically, the patient group already has a high risk of mortality (e.g., critically ill patients with pneumonia, people with mood disorders, and patients with HIV) [64, 65]; thus, the difference in RR for all‐cause mortality between PWUC and cannabis nonusers could be minimized. Another potential reason is that some patients may use cannabis for medical treatment to address their illness pain [66]. Therefore, significant RRs were found among the general population but not among the patient population in the present systematic review and meta‐analysis.

While our findings highlight potential risks associated with cannabis use, it is important to acknowledge the evolving landscape of cannabis research. Robust evidence supports therapeutic benefits of cannabinoids for specific medical conditions, including: chronic pain management (e.g., neuropathic pain), reducing chemotherapy‐induced nausea/vomiting, improving spasticity in multiple sclerosis, and treating rare forms of epilepsy (e.g., Dravet syndrome) [67, 68]. These clinical applications, primarily involving purified cannabinoids or standardized formulations under medical supervision, differ substantially from recreational use patterns examined in our study. The risk‐benefit profile appears highly context‐dependent, varying by: product composition (THC:CBD ratios), mode of administration, underlying patient conditions, and dosage regimens [69].

## 5 Strengths of the Study

This systematic review and meta‐analysis offers several important contributions to literature. First, it represents a comprehensive synthesis of evidence on cannabis use and all‐cause mortality, incorporating data from 14 cohort studies with over 17.5 million participants which is a sample size providing robust statistical power. Second, the analysis adhered rigorously to PRISMA guidelines, employing dual independent review at all stages and prospective protocol registration to minimize bias. Third, we identified critical effect modifiers through pre‐specified subgroup analyses (e.g., population type, study design) that explain between‐study heterogeneity. Fourth, all included studies demonstrated good methodological quality (Newcastle‐Ottawa Scale ≥ 5), with sensitivity analyses confirming result stability. Finally, the finding of consistent risk elevation across diverse study designs and geographic settings strengthens the evidence for an association, even as the exact mechanisms require further clarification.

## 6 Limitations of the Study

The present systematic review and meta‐analysis has several limitations. First, the lack of granular data on cannabis use patterns (e.g., frequency, potency, administration methods) in included studies limited dose–response analyses. Second, all evidence came from high‐income countries (*n* = 14 studies), potentially restricting generalizability to regions with different use patterns or healthcare systems. Third, while all‐cause mortality provides comprehensive risk assessment, it may include unrelated deaths, though the consistent direction of effect across studies supports result robustness. Fourth, methodological weaknesses in primary studies included predominant reliance on self‐reported exposure (*n* = 6), undocumented baseline mortality status (*n* = 7), and inadequate follow‐up reporting (*n* = 7). Finally, while we excluded studies with substantial polysubstance use, most included cohorts (*n* = 11) lacked prospective toxicological confirmation of cannabis‐specific effects.

## 7 Conclusion

This systematic review and meta‐analysis of 14 cohort studies found a significant association between cannabis use and increased all‐cause mortality risk (RR = 1.53, 95% CI: 1.09–2.14), though this relationship might be moderated by several factors. The association attenuated when controlling for comorbidities and substance co‐use (RR = 1.24, 95% CI: 0.97–1.58), suggesting the observed risk reflects a combination of potential pharmacological effects, pre‐existing health vulnerabilities, and socioeconomic confounders rather than cannabis‐specific causation. While these findings highlight cannabis use as a potential marker for elevated mortality risk, particularly in heavy users and those with psychiatric comorbidities, the exact mechanisms remain multifactorial and context dependent. Future research should employ causal inference methods to better isolate cannabis‐specific effects from concurrent risk factors.

## Author Contributions

**Zainab Alimoradi:** conceptualization, formal analysis, validation, investigation, writing – original draft, writing – review and editing, resources, visualization, methodology, software. **Chung‐Ying Lin:** validation, investigation, resources, writing – review and editing, writing – original draft, visualization. **Daniel T. Myran:** writing – review and editing, writing – original draft, data curation. **Marco Solmi:** writing – review and editing, visualization. **Amir H. Pakpour:** conceptualization, validation, formal analysis, investigation, resources, writing – review and editing, writing – original draft, visualization, supervision, project administration, methodology. All authors have read and approved the final version of the manuscript. Amir H. Pakpour had full access to all the data in this study and takes complete responsibility for the integrity of the data and the accuracy of the data analysis.

## Ethics Statement

The authors have nothing to report.

## Consent

The authors have nothing to report.

## Conflicts of Interest

M.S. received honoraria/has been a consultant for AbbVie, Angelini, Lundbeck, Otsuka. The other authors declare no conflicts of interest.

## Transparency Statement

Amir H Pakpour affirms that this manuscript is an honest, accurate, and transparent account of the study being reported; that no important aspects of the study have been omitted; and that any discrepancies from the study as planned and registered, have been explained.

## Supporting information

Supplement 1.

## Data Availability

The data that support the findings of this study are available from the corresponding author upon reasonable request.
