# Supplementary material for: Relative Risk of All‐Cause Mortality Associated With Cannabis Use: A Systematic Review and Meta‐Analysis of Cohort Studies
Source: Health Sci Rep. 2025 Sep 26;8(10):e71212. doi: 10.1002/hsr2.71212 (PMC12464731; doi:10.1002/hsr2.71212)
Supplement: Supplementary file 1 — Supplement 1. [file HSR2-8-e71212-s001.docx]

**Search syntax for PubMed:**

((cannabi* OR marijua* OR hashish* OR "Cannabis sativa" OR "Cannabis indica")

AND (mortalit* OR death* OR "all-cause mortality" OR "survival analysis"))
